# Interleukin-10 Promotes Pathological Angiogenesis by Regulating Macrophage Response to Hypoxia during Development

**DOI:** 10.1371/journal.pone.0003381

**Published:** 2008-10-13

**Authors:** Dru S. Dace, Aslam A. Khan, Jennifer Kelly, Rajendra S. Apte

**Affiliations:** 1 Department of Ophthalmology and Visual Sciences, Washington University School of Medicine, St. Louis, Missouri, United States of America; 2 Developmental Biology Program, Washington University School of Medicine, St. Louis, Missouri, United States of America; Instituto Oswaldo Cruz and FIOCRUZ, Brazil

## Abstract

Aberrant angiogenesis in the eye is the most common cause of blindness. The current study examined the role of interleukin-10 (IL-10) in ischemia-induced pathological angiogenesis called neovascularization during postnatal development. IL-10 deficiency resulted in significantly reduced pathological retinal angiogenesis. In contrast to the choroicapillaris where IL-10 interferes with macrophage influx, IL-10 did not prevent anti-angiogenic macrophages from migrating to the retina in response to hypoxia. Instead, IL-10 promoted retinal angiogenesis by altering macrophage angiogenic function, as macrophages from wild-type mice demonstrated increased vascular endothelial growth factor (VEGF) and nitric oxide (NO) compared to IL-10 deficient macrophages. IL-10 appears to directly affect macrophage responsiveness to hypoxia, as macrophages responded to hypoxia with increased levels of IL-10 and STAT3 phosphorylation as opposed to IL-10 deficient macrophages. Also, IL-10 deficient macrophages inhibited the proliferation of vascular endothelial cells in response to hypoxia while wild-type macrophages failed to do so. These findings suggest that hypoxia guides macrophage behavior to a pro-angiogenic phenotype via IL-10 activated pathways.

## Introduction

Angiogenesis is a critical process in maintaining tissue homeostasis during numerous physiological functions, such as wound-healing, reproduction, and embryonic development. However, unbridled angiogenesis can result in fulminant host disease. Abnormal angiogenesis is critical to the pathophysiology of diverse disease processes such as atherosclerotic heart disease and several cancers [1], [2], [3].

In the eye, this becomes especially important as abnormal angiogenesis (neovascularization) leads to blindness in several disease processes. Intraocular neovascularization, as characterized by abnormal retinal or choroidal angiogenesis, is a major cause of decreased vision in patients with diseases such as proliferative diabetic retinopathy (PDR): the leading cause of blindness in working adults, age-related macular degeneration (AMD): the leading cause of blindness in the elderly, and retinopathy of prematurity (ROP): the leading cause of blindness in premature infants [4], 5. In diabetic retinopathy, retinal neovascularization occurs in up to 20% of patients with diabetes [6]. Current laser ablation treatment for PDR has changed little over the 50 years since its first inception, and is applied only after onset of neovascularization. Although it reduces the risk of severe vision loss, laser photocoagulation reduces peripheral and night vision, and is uncomfortable and expensive [7]. There is recent evidence that the pathobiology of PDR is more complex. Immunological mechanisms, including exudation, upregulation of inflammatory mediators, and immune cell infiltration have been implicated in PDR [8]. Retinopathy of prematurity blinds 50,000 newborn babies annually worldwide. Peripheral retinal ischemia and the cessation of normal retinal vessel growth leads to compensatory angiogenesis, tractional retinal detachment, and blindness. Although diseases resulting in ocular neovascularization differ in many aspects, it is believed that tissue ischemia is the underlying cause leading to compensatory angiogenesis. Tissue ischemia can also result in cellular inflammation, including the infiltration of macrophages to the site of ischemia.

Macrophages carry out a wide variety of biological functions, including participation in neovascularization [9]. Macrophages can exhibit both pro-angiogenic and anti-angiogenic functions. This dual function of macrophages seems to be largely dependent upon the polarization of macrophages. Polarization, in turn, seems to be regulated by the production of cytokines in the resident tissue micro-milieu [10], [11], [12], [13]. Macrophages stimulated in the presence of interferon gamma (IFN-γ), lipopolysaccharide (LPS), or granulocyte macrophage colony-stimulating factor (GM-CSF) produce high levels IL-12, IL-23, IL-6, and tumor necrosis factor alpha (TNF-α), and low levels of IL-10. This “classically-activated” macrophage, or M1 macrophage, displays an anti-angiogenic phenotype, and plays an important role in anti-bacterial and pro-inflammatory functions. Macrophages stimulated in the presence of IL-10, IL-4, or IL-13 produce high levels of IL-10 and low levels of pro-inflammatory cytokines such as IL-6 and TNF-α. These “alternatively-activated” macrophages, or M2 macrophages, are pro-angiogenic. Of these cytokines, IL-10 may possess the most significant influence on the polarization of macrophages and their ability to regulate angiogenesis in the eye [10], [14].

AMD is a disease of the elderly characterized by blindness that is secondary to post-developmental choroidal angiogenesis. Termed choroidal neovascularization (CNV), this aberrant ocular angiogenesis develops in senescent tissues. In a mouse model of CNV, it has been shown that IL-10 promotes CNV by preventing macrophage infiltration into the choroid [14]. As the eye ages, IL-10 gene expression is upregulated, resulting in increased CNV in senescent tissues due to the ability of IL-10 to polarize macrophages towards a pro-angiogenic phenotype [10]. Macrophages also seem to be involved in PDR, as macrophages have been identified in the vitreous humor of diabetic patients [15], and have also been found in epiretinal membranes surgically removed from the eyes of diabetic patients [16]. In this study, we sought to determine if IL-10 affects murine retinal neovascularization during postnatal development, the cause of blindness in infants with ROP.

## Results

### IL-10^−/−^ mice demonstrate significantly reduced retinal neovascularization in response to ischemia

**Figure 1:**
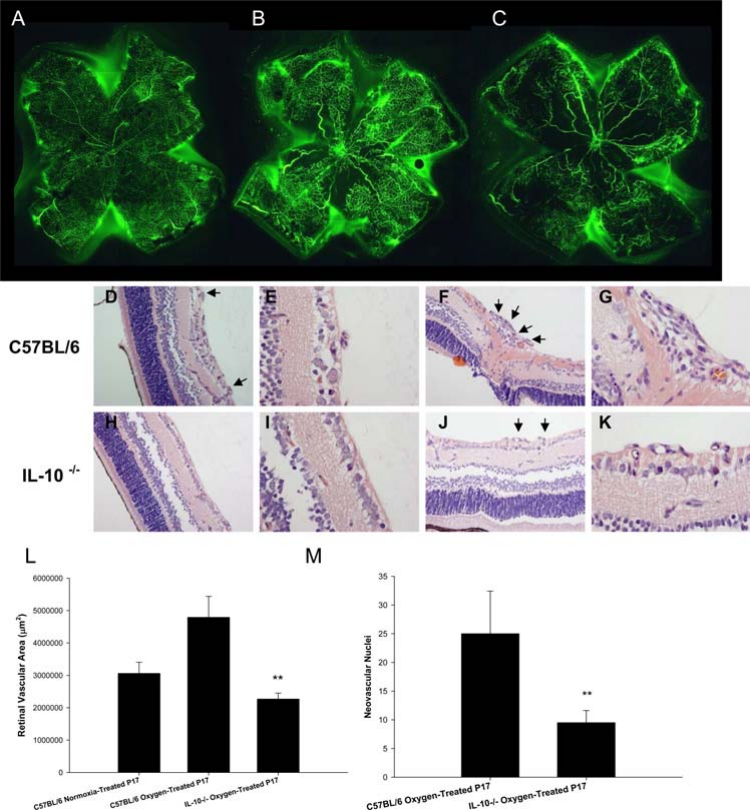
IL-10^−/−^ mice demonstrate significantly reduced retinal neovascularization in response to ischemia. C57BL/6 and IL-10^−/−^ were exposed to 75%±2% O_2_ from day P7 to P12. Mice were returned to normoxic conditions for 5 days, and on P17 animals were perfused with FITC-dextran, eyes harvested, and retina flatmounts made. Fluorescent microscopy of perfused retinas of (A) normoxic-treated wild-type mice (n = 5), (B) oxygen-treated wild-type mice (n = 5), and (C) oxygen-treated IL-10^−/−^ mice (n = 4) reveal decreased angiogenesis and increased areas of non-perfusion in IL-10^−/−^ mice. This experiment was repeated 2 additional times with similar results (D–G) H&E staining of ocular tissue sections from C57BL/6 oxygen-treated mice exhibit extensive preretinal neovascular loops (arrows), whereas (H–K) ocular tissue sections from IL-10^−/−^ oxygen-treated mice demonstrate a significant reduction in preretinal neovascular loops. Images were acquired at both 40× (D,F,H,J) and 100× (E,G,I,K). (L) The total area of retinal vascularization was quantified using Metamorph™ software as described in Materials and Methods, and plotted as a bar graph. IL-10^−/−^ mice exposed to oxygen had significantly reduced (**P = 0.0006) retinal vascularization compared to wild-type mice exposed to oxygen. (M) A bar graph represents the mean±SD number of neovascular loops of 10 separate sections, with IL-10^−/−^ mice demonstrating reduced neovascular loops (**P = 0.0071) compared to wild-type mice following OIR.

In order to determine if IL-10 affects developmental angiogenesis in the retina, we utilized the oxygen-induced retinopathy (OIR) model to induce tissue ischemia and compensatory retinal neovascularization [17]. Newborn C57BL/6 and IL-10^−/−^ pups were exposed to 75% oxygen for 5 days, between P7 and P12, and then returned to normal air conditions. The initial exposure to high oxygen levels causes central retinal vascular growth to slow or cease completely, and also causes developed retinal vessels to regress. As the pup then matures in a normoxic environment, the non-vascularized retina becomes increasingly metabolically active. The absence of adequate vascularization leads to tissue ischemia and hypoxia. This results in compensatory retinal neovascularization. To measure retinal neovascularization, P17 animals were perfused with FITC-dextran, eyes were harvested, and retinal flatmounts were made. We found that compared to pups that have never been subjected to high oxygen (Figure 1A), age-matched oxygen-treated C57BL/6 mice (Figure 1B) demonstrated significantly more neovascularization with an abundance of new blood vessel tufts, a central loss of normal retinal blood vessels, and increased retinal vascular tortuosity. Oxygen-treated IL-10^−/−^ mice (Figure 1C), however, demonstrated not only significantly reduced neovascularization, but also increased areas of ischemia and nonperfusion that lacked any vasculature. The total area of retinal vascularization was quantified by Metamorph™ software, and oxygen-treated IL-10^−/−^ mice demonstrated significantly less retinal vascularization compared to normoxic and hyperoxic wild-type mice (Figure 1L). Differences in retinal vasculature between wild-type and IL-10^−/−^ mice occur only following exposure to oxygen, as P17 normoxic IL-10^−/−^ retinas show no vascular differences compared to normoxic wild-type P17 retinas (data not shown).

In addition to retinal flatmounts, eyes from oxygen-treated mice were harvested for histological examination. Eyes were fixed in formalin and 4-µm sections were made and stained with hematoxylin and eosin. The neovascularization was assessed histologically by counting the number of vascular endothelial cell nuclei vitreal to the inner-limiting membrane. The retinas of oxygen-treated wild-type mice contained multiple neovascular tufts extending into the vitreous (Figures 1D–1G), whereas retinas from oxygen-treated IL-10^−/−^ mice had significantly fewer vascular endothelial cells at these locations (Figures 1H–1K). The neovascularization was quantified by counting and averaging 5 separate sections, revealing a significantly higher quantity of neovascular cell nuclei in the oxygen-treated wild-type mice compared to oxygen-treated IL-10^−/−^ mice (Figure 1M).

### Anti-IL-10 immunotherapy results in significantly reduced retinal neovascularization in response to hypoxia

**Figure 2:**
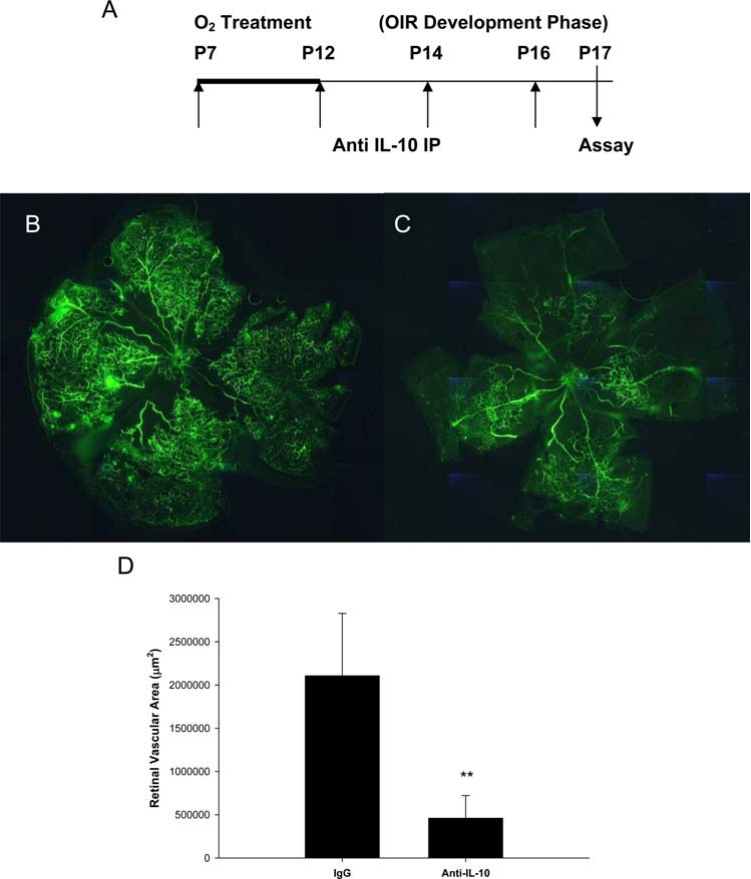
Anti-IL-10 immunotherapy results in significantly reduced retinal neovascularization in response to ischemia. (A) The experimental scheme for the treatment of C57BL/6 mice with either rat anti-mouse IL-10 blocking antibodies (n = 4) or rat IgG isotype (n = 6) control antibodies (150 µg) immediately before exposure to oxygen (day P7), immediately after exposure to oxygen (day P12), and 2 timepoints midway through secondary exposure to normoxia (days P14 and P16). On day P17 animals were perfused with FITC-dextran, eyes were harvested, and retinal flatmounts were made. Fluorescent angiography of (B) isotype control treated mice and (C) anti-IL-10 treated mice reveal significantly decreased angiogenesis and increased areas of non-perfusion in anti-IL-10 treated mice following oxygen treatment. (D) The total area of retinal vascularization was quantified using Metamorph™ software as described in Materials and Methods and plotted as a bar graph. Anti-IL-10 treated mice had significantly reduced retinal vascularization (**P = 0.0052) compared to IgG treated mice. This experiment was repeated with similar results.

Since we observed less retinal neovascularization in IL-10^−/−^ mice, we sought to determine if IL-10 may be an ideal therapeutic target for the treatment of aberrant retinal angiogenesis. Therefore, we administered blocking antibodies against murine IL-10 in wild-type mice. Anti-IL-10 or IgG antibodies (150 µg) were injected into wild-type mice immediately before exposure to oxygen (day P7), immediately following exposure to oxygen (day P12), and midway between secondary normoxia exposure (days P14 and P16) (Figure 2A). On day P17 mice were perfused with FITC-dextran and retinal flatmounts were made. Fluorescein angiography revealed that compared to IgG treated mice (Figure 2B), mice treated with anti-IL-10 blocking antibodies developed reduced retinal neovascularization following OIR (Figure 2C). Analysis of retinal flatmounts with Metamorph™ software revealed a statistically significant reduction in retinal vascularization (Figure 2D). This suggests that anti-IL-10 immunotherapy may be an attractive avenue for the treatment of retinopathy of prematurity.

### IL-10 does not alter infiltration of F4/80^+^ macrophages into ischemic retinas, but leads to significantly increased production of pro-angiogenic NO

**Figure 3:**
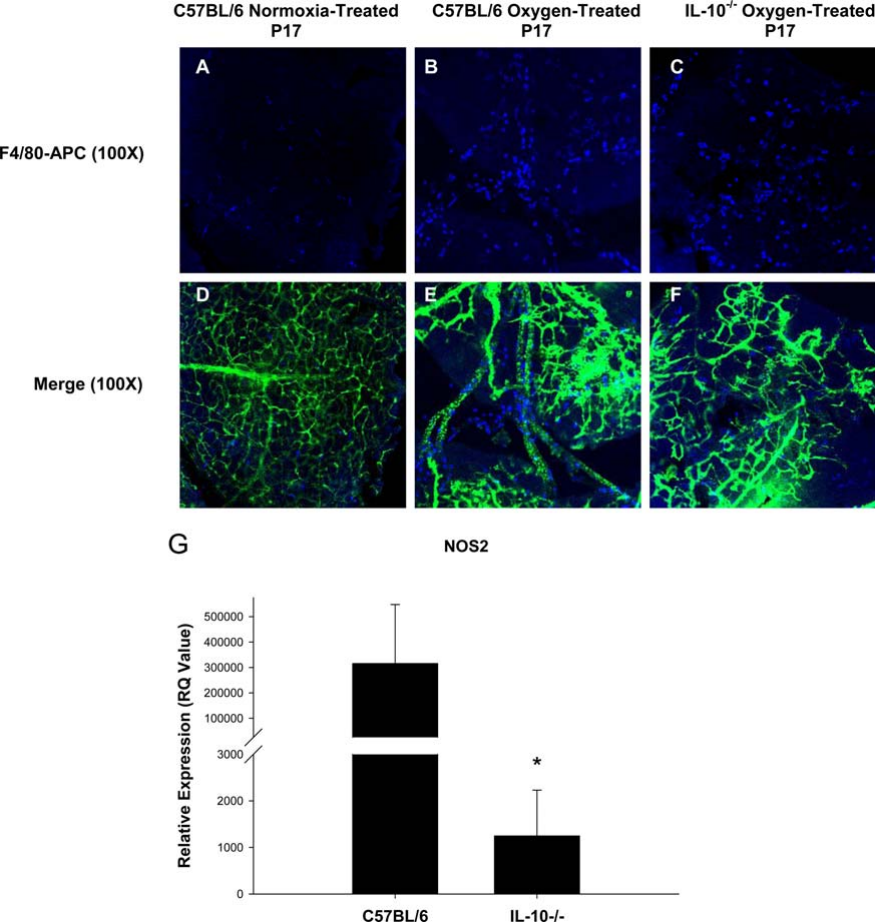
F4/80^+^ macrophages infiltrate ischemic retinas of both C57BL/6 and IL-10^−/−^ mice. Retinal flatmounts of P17, FITC-dextran perfused C57BL/6 normoxia-treated, C57BL/6 oxygen-treated, and IL-10^−/−^ oxygen-treated retinas were stained with Allophycocyanin (APC) anti-mouse F4/80 antibody. In contrast to the paucity of cells in the (A) normoxic wild-type retina, a significant number of F4/80^+^ macrophages are observed in the retinas of both (B) C57BL/6 oxygen-treated and (C) IL-10^−/−^ oxygen-treated mice. (D, E, F) Examining both macrophage infiltrate and blood vessels reveals that APC-labeled macrophages reside primarily along FITC-dextran perfused blood vessels. Since IL-10 did not prevent macrophage infiltration into the retina, we examined the genetic profile of retinal macrophages. Using splenic wild-type macrophages as a baseline, retinal macrophages from wild-type mice exhibit significantly (p<0.05) increased expression of the pro-angiogenic gene (G) nitric oxide compared to IL-10^−/−^ retinal macrophages following OIR, suggesting that IL-10 promotes angiogenesis by polarizing macrophages towards a pro-angiogenic phenotype.

It has been previously shown that macrophages infiltrate the retinas of mice following OIR [18], [19], [20]. Our laboratory has shown that IL-10 prevents the infiltration of macrophages into the choroidal vasculature of the eye in murine models of CNV [14]. We sought to determine if IL-10 prevents the infiltration of macrophages into a distinct vascular plexus of the eye, the retina, following OIR. We stained FITC-perfused retinal flatmounts with an allophycocyanin (APC)-labeled anti-mouse F4/80 antibody to detect the presence of infiltrating macrophages. Wild-type normoxia retinas had very few F4/80^+^ cells (Figure 3A), which might not be infiltrating macrophages but rather resident F4/80^+^ retinal microglia [19]. As expected, retinas from oxygen-treated C57BL/6 mice displayed a significant increase in the number of infiltrating F4/80^+^ macrophages compared to retinas from normoxic control mice (Figure 3B). Surprisingly, retinas from oxygen-treated IL-10^−/−^ mice did not display an increased number of F4/80^+^ macrophages compared to retinas from wild-type oxygen-treated mice, as similar numbers of F4/80+ cells are observed (Figure 3C). Therefore, the lack of neovascularization observed in IL-10^−/−^ mice following OIR is not simply due to an increased influx of anti-angiogenic F4/80^+^ macrophages infiltrating the retina. Interestingly, infiltrating F4/80^+^ macrophages in both groups of oxygen-treated mice resided along FITC-dextran labeled blood vessels (Figures 3E and 3F), supporting previous work that has observed infiltrating macrophages aligning along neovascular tufts following OIR and suggesting macrophages may modulate vessel growth and regression [19], [20].

Since IL-10 did not alter the infiltration of macrophages into the retina following OIR, we hypothesized that IL-10 may be altering the angiogenic phenotype of macrophages in the local tissues, as seen in the senescent choroid during CNV in the eye and in systemic tumors [10], [21]. To determine if retinal macrophages from wild-type and IL-10^−/−^ mice were exhibiting differential angiogenic phenotypes, we examined the gene expression profiles of *in vivo* derived retinal macrophages following oxygen-induced retinopathy. Retinal macrophages were isolated from retinal tissues by density gradient centrifugation. Following RNA isolation and cDNA preparation, several genes were examined, including IL-12, IL-23, IL-1, TNF-α, arginase (Arg1), transforming growth factor beta (TGF-β) (data not shown) and nitric oxide synthase (NOS2). Of all the genes tested, a significant difference between wild-type and IL-10 deficient retinal macrophages was observed only in NOS2, the gene responsible for macrophage nitric oxide (NO) production. Using splenic C57BL/6 F4/80^+^ macrophages as a baseline, gene expression of NOS2 was found to be significantly higher in wild-type C57BL/6 retinal macrophages (Figure 3G). NO is involved in multiple host processes such as neurotransmission, vasodilation, host defense, and inflammation [22], [23]. NO is also known to influence neovascularization, and its effect can be either pro-angiogenic [24], [25] or anti-angiogenic [26]. These opposing effects of NO on angiogenesis may be dependent upon the local microenvironment. Of potential interest is previous work that has shown that in the retina, nitric oxide is proangiogenic [27]. In their study, Ando et al. found that in a VEGF transgenic mouse model, deficiency of any of the three isoforms of NO (eNOS, iNOS, and nNOS) resulted in a decrease in subretinal neovascularization at P21 during normal development [27]. This suggests that not only is NO proangiogenic in the retina, but that regulation of VEGF driven angiogenesis involves NO.

### IL-10 and hypoxia skew macrophages toward a pro-angiogenic phenotype

**Figure 4:**
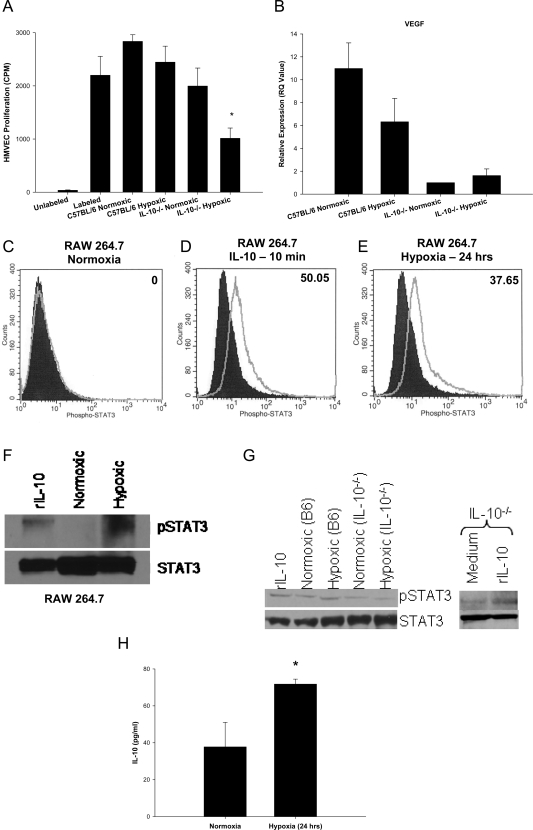
Macrophage responses to hypoxia. Splenic F4/80^+^ macrophages from IL-10^−/−^ mice (A) significantly inhibit (*P = 0.0163) the proliferation of HMVECs following exposure to hypoxia compared to hypoxia-treated wild-type macrophages. Splenic F4/80^+^ macrophages from wild-type mice do not significantly (P>0.05) inhibit HMVEC proliferation compared to untreated HMVECs. This could be due to (B) increased expression of VEGF in wild-type macrophages compared to IL-10^−/−^ macrophages. The IL-10 signaling pathway appears to be activated following exposure to hypoxia. RAW 264.7 murine macrophages (C) do not exhibit increased levels of phosphorylation of STAT3 under normoxic conditions (open histogram) compared to IgG stained cells (shaded histogram). However, (E) exposure to hypoxia (open histogram) results in a significant increase in phosphorylation of STAT3 compared to normoxia-treated cells (shaded histogram). This increase in phosphorylation of STAT3 is at levels similar to (D) RAW 264.7 cells exposed to recombinant IL-10 protein for 10 min (open histogram) versus normoxic cells (shaded histogram). Inset numbers indicate percentage of positive cells above normoxic controls. Phosphorylation of STAT3 protein following hypoxia in RAW 264.7 cells was also confirmed with (F) western blot analysis. Phosphorylation of STAT3 occurred following exposure to both recombinant IL-10 protein for 10 min and a 24 hour exposure to hypoxia. Probing of total STAT3 protein was used as a loading control. These findings in RAW macrophages were also observed in primary macrophages (G). Wild-type macrophages treated with recombinant IL-10 protein for 10 minutes or hypoxia for 24 hours demonstrated increased phosphorylation of STAT3 compared to baseline normoxic levels. IL-10^−/−^ macrophages, however, demonstrated decreased levels of pSTAT3 at baseline normoxic levels compared to wild-type normoxic macrophages, and did not upregulate pSTAT3 in response to hypoxia. STAT3 signaling is still intact in IL-10^−/−^ macrophages, as phosphorylation of STAT3 in increased in IL-10^−/−^ macrophages following stimulation with recombinant IL-10 protein. This increase in STAT3 phosphorylation may be due to the (H) significantly increased (*P = 0.0123) production of IL-10 protein by RAW 264.7 macrophages following exposure to hypoxia compared to normoxia-treated RAW macrophages.

Within the retinas of mice exposed to oxygen-induced retinopathy, there is local tissue hypoxia leading to compensatory angiogenesis. Since macrophages migrate into the hypoxic environment of the retina, we sought to determine the anti-angiogenic behavior of macrophages in response to hypoxia. We developed an *in vitro* assay where F4/80^+^ macrophages were isolated from the spleens of wild-type C57BL/6 and IL-10^−/−^ mice by magnetic bead isolation. F4/80^+^ cells were exposed to either normoxic (5% CO_2_, 21% O_2_) or hypoxic (5% CO_2_, 4% O_2_) conditions. Following a 24 hour exposure to such conditions, macrophages were then cocultured with human dermal microvascular endothelial (HMVEC) cells. HMVEC cell proliferation was then assessed by [^3^H] thymidine incorporation. We have previously demonstrated that the anti-angiogenic properties of mouse macrophages could be assessed by coculture with HMVEC cells [10]. Interestingly, IL-10 deficient macrophages exposed to hypoxic conditions significantly inhibited the proliferation of HMVEC cells, while hypoxic wild-type C57BL/6 macrophages failed to do so (Figure 4A). This inhibition of HMVEC proliferation was not observed in normoxic wild-type C57BL/6 macrophages (neither normoxic nor hypoxic-treated) or in normoxic IL-10^−/−^ macrophages. The addition of recombinant IL-10 protein during hypoxic treatment of IL-10^−/−^ macrophages did not result in the restoration of HMVEC proliferation (data not shown), suggesting that IL-10 may be needed during the development and differentiation of macrophages for a pro-angiogenic phenotype to occur.

Vascular endothelial growth factor (VEGF), which increases microvascular permeability and is a specific mitogen for endothelial cells, is a pro-angiogenic factor expressed by macrophages [28]. The importance of VEGF in OIR has been shown previously, as inhibition of VEGF results in a significant reduction of retinal neovascularization [29]. Therefore, we examined VEGF gene expression in C57BL/6 and IL-10^−/−^ macrophages following a 24 hour exposure to normoxia or hypoxia. In both conditions, VEGF gene expression was significantly higher in wild-type macrophages compared to IL-10^−/−^ macrophages (Figure 4B). This may partly explain why C57BL/6 macrophages failed to inhibit the proliferation of HMVEC cells *in vitro*, and why wild-type macrophages promote the development of retinal and pre-retinal neovascularization in OIR *in vivo* while IL-10^−/−^ macrophages failed to do so despite infiltrating the retina in numbers equal to wild-type mice. This pattern of VEGF expression is restricted to macrophages, as VEGF in whole retinal tissue from oxygen-treated wild-type mice was not significantly elevated compared to normoxic and oxygen-treated IL-10^−/−^ whole retinal tissue (data not shown). Therefore, the localized expression of VEGF on macrophages may be influencing the development of retinal blood vessels. This correlates with the work of Naug et al, who found VEGF-expressing macrophages associated with vitreal loops in wild-type mice following OIR [30].

We next examined whether hypoxia induced IL-10 signaling in immunocompetent wild-type macrophages. IL-10 protein binds to the IL-10 receptor (IL-10R), resulting in phosphorylation and activation of the signal transducer and activator of transcription 3 (STAT3). STAT3 phosphorylation and activation is essential for all known function of IL-10 [31]. To determine if immunocompetent macrophages were undergoing IL-10 signaling, RAW 264.7 murine macrophages were assessed for phosphorylation of STAT3 by flow cytometry and western blot analysis. RAW macrophages cultured in normoxic conditions (21% O_2_) did not demonstrate increased staining of phospho-STAT3 antibody compared to isotype control antibody stained cells (Figure 4C). RAW macrophages pulsed with recombinant IL-10 protein (100 ng/ml) for 10 minutes demonstrated a significant increase in phospho-STAT3 staining compared to normoxic treated cells (Figure 4D), confirming that IL-10 signaling in macrophages results in phosphorylation of STAT3. RAW macrophages cultured in hypoxic conditions (4% O_2_) for 24 hours also demonstrated a significant increase in phospho-STAT3 staining compared to normoxic treated cells (Figure 4E). This suggests that when immunocompetent macrophages infiltrate hypoxic regions such as the retina in OIR, the IL-10 signaling pathway is induced. Flow cytometric analysis of STAT3 phosphorylation was confirmed by western blot examination (Figure 4F). RAW macrophages treated with either recombinant IL-10 protein (100 ng/ml) for 10 minutes or hypoxia for 24 hours demonstrated increased phosphorylation of STAT3. These findings in RAW macrophages were also observed in primary macrophages. Wild-type macrophages treated with IL-10 protein for 10 minutes or hypoxia for 24 hours demonstrated increased phosphorylation of STAT3 compared to baseline normoxic levels (Figure 4G). IL-10^−/−^ macrophages, however, demonstrated decreased levels of pSTAT3 at baseline normoxic levels compared to wild-type normoxic macrophages, and did not upregulate pSTAT3 in response to hypoxia. STAT3 signaling is still intact in IL-10^−/−^ macrophages, as phosphorylation of STAT3 is increased in IL-10^−/−^ macrophages following stimulation with recombinant IL-10 protein (Figure 4G). Interestingly, supernatants from RAW macrophages cultured under hypoxic conditions demonstrate a significant increase in the production of IL-10 protein (Figure 4H). Therefore, phosphorylation of STAT-3 in macrophages following hypoxia may be due to autocrine secretion of IL-10 by macrophages in response to hypoxia and subsequent engagement of the IL-10 receptor. It has been shown that STAT3 activation and signaling can result in the expression of VEGF [32], correlating with our earlier observation of increased VEGF gene expression levels in immunocompetent macrophages.

## Discussion

In summary, IL-10 promotes retinal neovascularization in a mouse model of oxygen-induced retinopathy. In contrast to previous reports that analyzed the regulation of choroidal macrophages by IL-10 [14], IL-10 did not prevent the infiltration of macrophages into the retina, but rather polarized the macrophage genetic profile towards a pro-angiogenic phenotype. This difference in IL-10 function may be attributable to the local tissue micro milieu, as the hypoxic drive and regional soluble and membrane-bound signals in diverse vascular beds may differ, i.e. the retinal vasculature versus the choriocapillaris. Although IL-10 promotes angiogenesis by preventing the infiltration of anti-angiogenic macrophages into the choroid [14], this study suggests that in the retina, IL-10 promotes angiogenesis by directly polarizing macrophages towards a pro-angiogenic phenotype. Unlike choroidal neovascularization, hypoxia or ischemia is the prime driver of retinal and pre-retinal neovascularization observed in diabetic retinopathy and ROP. Hypoxia induces a profound change in the phenotype of macrophages. A recent study demonstrated that more than 30 pro-angiogenic genes were upregulated by hypoxia in primary macrophages [33], including VEGF and inducible nitric oxide synthase (iNOS). Most of the work on hypoxia and macrophages has focused on tumor-associated macrophages (TAMs). Macrophages have been shown to accumulate in hypoxic regions of tumors, and secrete multiple growth factors and pro-angiogenic cytokines that promote tumor growth, angiogenesis, metastasis, and immune evasion [34]. To our knowledge, this is the first study that reveals that hypoxia and IL-10 function synergistically to influence macrophage function and angiogenic homeostasis. It is also noteworthy that the pro-angiogenic effects of hypoxic macrophages are IL-10 dependent and require IL-10 mediated signal transduction.

The diverse and complex regulation of angiogenesis by IL-10 is highlighted by the finding that in a mouse model of post-developmental surgically-induced hindlimb ischemia, IL-10 appears to function in an anti-angiogenic manner, and does so by downregulating VEGF expression [35]. These findings illustrate the multifaceted role of IL-10 in regulating angiogenesis. The vascular bed and the tissue type likely have a profound impact on the ability of IL-10 to regulate macrophage behavior after tissue injury. The success of anti-IL-10 immunotherapy in our studies of mice during OIR was achieved by systemic depletion of IL-10 by intraperitoneal injection. If IL-10 immunotherapy is to be translated to humans as a potential treatment modality for retinopathy, local intraocular injection of anti-IL-10 antibodies or other IL-10 inhibitors, similar to anti-VEGF injections for AMD patients, may be desirable to minimize the systemic effects in non-ocular vascular beds.

The role of IL-10 in the induction of developmental angiogenesis seems to be a highly choreographed process involving multiple factors. Interestingly, all of the genes that were upregulated in macrophages following oxygen-induced retinopathy were found to be 1) significantly upregulated in C57BL/6 macrophages and not in IL-10 deficient macrophages, 2) proangiogenic, and 3) cross regulative. IL-10 has previously been shown to upregulate NO production in activated macrophages in a dose dependent manner [36]. Also, nitric oxide has previously been reported to be upregulated in hypoxic retinas [37]. Our findings demonstrate that this increase in NOS2 expression in the retina may be attributable to infiltrating retinal macrophages. The relationship between nitric oxide and VEGF is highly complex. In other models of hypoxia-induced angiogenesis, VEGF and NO were responsible for increased vascular permeability [38]. In the eye, transgenic mice with increased expression of VEGF in photoreceptors had decreased subretinal neovascularization when nitric oxide was deleted [27], suggesting that VEGF driven angiogenesis was NO dependent. It has also been shown in other cell types that hypoxia results in STAT3 phosphorylation, which increases cellular levels of hypoxia-inducible factor (HIF-1α) which in turn, increases VEGF expression [39]. Additionally, increased STAT3 and pSTAT3 levels have been found in the retina of mice following OIR[40].

The role of macrophages in preventing retinal angiogenesis appears to be by inhibiting the proliferation of vascular endothelium. Inhibition of vascular endothelial cell proliferation observed *in vitro* following co-culture with hypoxia-treated IL-10^−/−^ macrophages (Fig. 4A) may be attributable to multiple factors. Our previous work suggests that IL-10 deficient macrophages tend to skew towards a pro-inflammatory/anti-angiogenic phenotype (M1) following activation, whereas wild-type macrophages have a mixed pro/anti inflammatory phenotype [10], [14]. In this study, hypoxia is the stimulus that activates and skews IL-10^−/−^ macrophages towards an M1 phenotype. These M1 polarized IL-10^−/−^ macrophages likely inhibit HMVEC proliferation due to a deficiency of NO (Fig. 3G), or increased expression of FasL, as shown previously [14]. Under normoxic environments, IL-10^−/−^ macrophages are in an inactive, non-polarized state, and thus do not affect HMVEC proliferation.

Taken as a whole, inhibition of IL-10 signaling in macrophages may be beneficial in not only preventing aberrant angiogenesis in the eye, but may also be relevant in the prevention of tumor-infiltrating macrophages from becoming pro-angiogenic TAMs that have been implicated in tumor progression and metastasis.

## Materials and Methods

### Mice

C57BL/6 and IL-10^−/−^ (B6.129P2-*Il10^tm1Cgn^*/J) mice were purchased from the Jackson Laboratory (Bar Harbor, ME). All work was carried out in accordance with Association for Research in Vision and Ophthalmology (ARVO) guidelines. Comprehensive protocols of animal care and experimental design outlined in this study were approved by the Animal Studies Committee of Washington University.

### Oxygen-induced Retinopathy and Quantification of Neovascularization

Oxygen-induced retinopathy was produced in mice by the method described by Smith et al. [17]. On day 7 postpartum (P7), neonatal mice and their nursing mothers were exposed to 75%±2% oxygen for 5 days. On day P12, mice were then returned to room air for a period of 5 days. At day P17, mice were anesthetized with 0.66 mg/kg ketamine hydrochloride (Vetalar; Parke-Davis) given i.p. Mice were sacrificed by intracardiac perfusion with 2 ml of 20 mg/ml 2×10^6^ molecular weight FITC-dextran (Sigma-Aldrich, St. Louis, MO) in PBS. Eyes were enucleated, fixed in 4% paraformaldehye for 45 min at 4°C, and placed in PBS for 15 min at 4°C. A dissecting microscope was used to remove the cornea and lens and gently separate the retina from the underlying choroid and sclera. Microscissors were used to make four radial incisions of the retinal eyecup in order to prepare retinal flat mounts on glass slides. Flat mounts were immersed in Gel/Mount (Biomeda Corp., Foster City, CA) and coverslips were carefully placed. Retinal vascularization was analyzed by fluorescent microscopy (Leica LMD 6000 fluorescent microscope; Wetzlar, Germany). Separate images were taken at 63× magnification, and images were merged using the ‘specimen overview’ function of the microscope software. The extent of retinal vascularization was then quantified by Metamorph™ Imaging software (Universal Imaging, Sunnyvale, CA). Briefly, the entire retina was outlined to identify the total retinal area of each eye. Then, the color threshold of each image was adjusted to highlight only the FITC-perfused vessels of the inclusively highlighted retina. This allowed the determination of total blood vessel area of each retina and the percentage of each retina that is occupied with blood vessels. Data was reported as total retinal vascular area, and the vascular area for all eyes in a treatment group were averaged and compared individually to controls using Student's *t* test.

Retinal neovascularization was also examined by histology. The eyes of P17 mice (n≥3 per group) were enucleated, fixed in 10% formalin, and embedded in paraffin. Serial 4-µm-thick sections of the eye were obtained and stained with hematoxylin and eosin. Images were obtained at 40× and 100× magnification. All retinal vascular cell nuclei anterior to the internal-limiting membrane were counted. 10 sections were examined, and the average±SD of neovascular cell nuclei per sections was obtained. No vascular cell nuclei anterior to the internal-limiting membrane were observed in normoxic control animals.

### *In Vivo* Anti-IL-10 Antibody Treatment

Rat anti-mouse IL-10 (JES5.2A5; Genzyme) and isotype control antibody (Rat IgG; Sigma) were administered to wild-type mice by intraperitoneal (i.p.) injection. Antibodies (150 µg) were administered at days P7 (before oxygen exposure), P12 (after oxygen exposure), P14, and P16, following the same timeline used previously for the i.p. injections during OIR [41]. Retinal neovascularization was then evaluated on day P17 as described above.

### Staining of retinal macrophages

Retinas were also examined for infiltration of macrophages. Flat-mounts of FITC-dextran perfused retinas were prepared as described above. Retinas were washed with PBS and blocked with 3% BSA in PBS for 30 min at 37°C. Following a subsequent washing with PBS, retinas were stained with 1 µg/ml Allophycocyanin (APC)-conjugated anti-mouse F4/80 antibody (eBioscience, San Diego, CA) for 40 min at 37°C. Retinas were washed again with PBS, immersed in Gel/Mount (Biomeda Corp., Foster City, CA), coverslipped, and examined by confocal microscopy.

### *In vitro* Hypoxic Treatment of Macrophages

Spleens were isolated from wild-type C57BL/6 and IL-10^−/−^ mice. Single-cell suspensions were obtained and stained with 1 µg/ml PE-conjugated anti-mouse F4/80 (eBioscience). PE-labeled cells were isolated by positive selection using magnetic separation (Stemcell Technologies, Inc.). F4/80^+^ macrophages were then exposed to normoxic (5% CO_2_, 21% O_2_) or hypoxic (5% CO_2_, 4% O_2_) conditions at 37°C for 24 hours. Cells were either immediately lysed for RNA isolation or used in the vascular endothelial cell proliferation assay.

### Vascular endothelial cell proliferation assay

Human dermal microvascular endothelial cells (HMVECs) (Lonza, Walkersville, MD) (5×10^4^) in log phase growth were placed in EGM2V media (Lonza, Walkersville, MD) in 96-well round bottom plates and allowed to adhere for 24 hours at 37°C. Normoxic or hypoxic macrophages were then added to HMVECs and allowed to co-culture for an additional 12 hours. Macrophages were removed by repeated and gentle pipetting, and HMVECs were incubated with 40 µCi/ml of [^3^H] thymidine (TRA61- GE Health Care, Piscataway, NJ) for 12 hours. The tissue culture plate was harvested and read using a Topcount harvester and microplate reader (Packard, Meriden, CT). The amount of [^3^H] thymidine incorporated into the HMVEC cells is directly proportional to the proliferation of the cells.

### Isolation of Retinal Macrophages

Retinas from P17 oxygen-treated mice were isolated from the rest of the eye as described above and placed directly in 1 ml of a 1 µg/ml collagenase (Sigma)/PBS solution. Retinas were then passed through a 18-gauge needle to obtain a single-cell suspension, and then allowed to incubate in collagenase for 30 min at 37°C. The collagenase solution was then brought to a total volume of 5 ml, and layered on top of 3 ml of Histopaque-1077 (Sigma) solution. Cells were spun at 2000 RPM for 30 min. During centrifugation, mononuclear cells remain at the PBS-Histopaque interface. Cells were isolated from the interface and immediately lysed for RNA isolation.

### Quantitative Analysis of Gene Expression (Quantitative Real-Time RT-PCR)

Total RNA was prepared from retinal macrophages from P17 oxygen-treated C57BL/6 and IL-10^−/−^ mice using the RNeasy mini kit (Qiagen, Valencia, CA). RNA was purified by gDNA eliminator spin columns and subsequent DNase treatment in order to minimize the amount of gDNA cross-contamination. cDNA was prepared using the High Capacity cDNA Archive Kit (Applied Biosystems, Foster City, CA). Relative levels of target gene expression were measured on the 7500 Real-time RT-PCR system (Applied Biosystems). For fluorescence measure and assay preparation, specific FAM-490 based Taqman Gene Expression Assay Mix (Applied Biosystems) for each gene of interest and Taqman Universal Master Mix (Applied Biosystems) were used. Relative Quantification PCR analysis was performed using the ABI 7500 SDS Software. The threshold cycle (Ct) values were measured for our endogenous control (β-actin) and the genes of interest at the logarithmic phase of amplification. The ΔCt values for the calibrator (negative baseline control) were calculated as the difference between the Ct value of the endogenous control and the target gene of interest (Ct β-actin – Ct target). This ΔCt value for the calibrator was then compared to the ΔCt value of each of our unknown cDNA samples, the difference of these two values were taken to achieve the ΔΔCt value for each gene (ΔCt unknown – ΔCt calibrator). The relative fold expression difference was calculated and graphed using the formula: 2(^−ΔΔCt^).

Taqman Gene Expression Assay Mixes used (primer/probe sets) include β-actin (Mm00607939_s1); TNF-α (Mm00443258_m1); IL-6 (Mm00446190_m1); IL-12 (Mm01288989_m1); IL-23 (Mm00518984_m1); IL-10 (Mm00439616_m1); FasL (Mm00438864_m1); IL-1β (Mm00434228_m1); IFN-γ (Mm00801778_m1); TGF-β (Mm03024053_m1); Arg1 (Mm00475988_m1); NOS2 (Mm00440485_m1); and VEGF-α (Mm00437304_m1).

For RT-PCR of *in vitro* treated macrophages, IL-10^−/−^ normoxic macrophages were used as a baseline comparison. For *in vivo* retinal macrophages, splenic F4/80^+^ macrophages were used as a baseline comparison.

### Assessment of macrophages for phospho-STAT3 expression

RAW 264.7 murine macrophages (ATCC; Manassas, VA) or splenic F4/80^+^ cells from C57BL/6 or IL-10^−/−^ mice were placed in complete RPMI media and incubated either in normoxic (21% oxygen) or hypoxic (4% oxygen) conditions for 24 hours. For flow cytometric analysis, cells were fixed and permeabilized with BD Cytofix/Cytoperm (Becton Dickinson). Cells were then stained with either rabbit IgG (2 µg/ml; Sigma) or rabbit anti-phospho STAT3 (1∶500; Sigma), followed by FITC-conjugated goat anti-rabbit antibody (2 µg/ml; Sigma). Macrophages stimulated with recombinant murine IL-10 (100 ng/ml; R&D Systems) for 10 min were used as a positive control for staining. Cells were washed, resuspended in PBS, and analyzed using a FACScan flow cytometer (BD Biosciences). Results were analyzed using CellQuest v.3.1f software (BD Biosciences). For examination of STAT3 phosphorylation by western blot, normoxic and hypoxic macrophages were lysed with Triton X-100 buffer (150 mM NaCl, 20 mM 1% Triton X-100), run on SDS-PAGE (10% NuPAGE gel from Invitrogen, Carlsbad, CA), and transferred onto a nitrocellulose membrane (150 mAmp for 90 min). The membrane was incubated with 5% nonfat dry milk for 1 h and washed three times for 10 min each with TBS-T. The blot was then incubated either with anti-phospho STAT3 (Cell Signaling Tech., Danvers, MA) or anti-STAT3 (Sigma) and then washed with TBS-T three times. The blot was then incubated with the corresponding secondary antibody conjugated with HRP for 1 h, washed three times with TBS-T for 10 min each, and then developed using ECL reagent.

### IL-10 enzyme-linked immunosorbent assay (ELISA)

RAW 264.7 macrophages were incubated in either normoxic or hypoxic conditions as described above. Cell supernatants were then harvested and levels of IL-10 protein in cell supernatants were determined using a mouse IL-10 Quantikine ELISA kit (R&D Systems).

### Statistical analysis

Results are presented as mean±standard deviation. A Student's *t*-test was used to assess the statistical significance of the differences between compared groups. A *P*-value of <0.05 was considered significant.
